# Recovery of the Voluntary Hospitals

**Published:** 1924-11

**Authors:** 


### RECOVERY OF THE VOLUNTARY HOSPITALS.

### FIRST POST-WAR SURPLUS.

The Fifth Annual Report of the Joint Council of the Order of St. John and the British Red Cross on the provincial hospitals of Great Britain has been issued at the price of 1s. 6d. (19, Berkeley Street, W.). It is the first report by the new Director, Dr. Kay Menzies, who succeeded the late Sir Napier Burnett only this year. The report reviews 710 hospitals in Great Britain possessing 43,404 beds, leaving unreviewed 45 hospitals who have not responded containing 999 beds. In the London area, which is outside the scope of the report, there are 116 hospitals with 13,002 available beds, so that in Great Britain there are 871 voluntary hospitals with 57,405 available beds.

### Financial Survey.

The following is a general summary of the finances of the hospitals reviewed in 1923 :—

| | £ | £ | | £ | £ |
|---|---|---|---|---|---|
| *England and Wales.* | | | | | |
| Ordinary Income .. | 4,620,769 | | Ordinary Expenditure | 4,351.057 | |
| Extraordinary Income and Receipts for Capital Purposes .. | 1,764,016 | | Extraordinary and Capital Expenditure | 1,102,665 | |
| | | £6,384,785 | | | £5,453,722 |
| *Scotland.* | | | | | |
| Ordinary Income .. | 765,547 | | Ordinary Expenditure | 821,565 | |
| Extraordinary Income and Receipts for Capital Purposes .. | 538,740 | | Extraordinary and Capital Expenditure | 105,828 | |
| | | 1,304,287 | | | 927,393 |
| Total Receipts .. | | £7,689,072 | Total Expenditure | | £6,381,115 |

These figures give some indication of the magnitude of the voluntary system. Of the total receipts, £7,051,248 was raised by voluntary effort, the remainder, £637,824 (8.29 per cent. of the total), being derived from public services, £550,918, and grants from the Voluntary Hospitals Commission, £86,906. They show that the ordinary income exceeded the ordinary expenditure by £213,694, and that the extraordinary income and receipts for capital purposes exceeded the extraordinary and capital expenditure by £1,094,263.

### "Ordinary" and "Extraordinary."

In order to make the financial position of the provincial voluntary hospitals still clearer, an explanation of the terms "ordinary" and "extraordinary" as used in hospital finance is added: "A very careful analysis of all the sources from which a hospital draws its financial support brings out more and more clearly each year this important point: That to obtain an accurate idea of the ability of an individual hospital and of the hospitals as a whole to find money to meet their maintenance costs each year, it is necessary to take into account something more than those sums of money which in hospital book-keeping nomenclature are classified as ordinary income." What the public want to know is the actual yearly cost of maintenance and the amount of money received each year for meeting that cost. A large proportion of "extraordinary income" is available for that purpose, and many hospitals showing a deficit on ordinary income may show a surplus for maintenance purposes when extraordinary income is taken into account. It may be noted that if the figures of the ordinary income and the ordinary expenditure of the Scottish hospitals alone are studied, it might be inferred that, as a whole, they were unable to meet their running costs during 1923, whereas the reverse is the case, for there was available out of extraordinary income a sum which was more than sufficient to make good any deficiency of ordinary income—a sum which it would be quite reasonable to use for this purpose.

### Investments and Interest.

The invested funds of the hospitals in England and Wales for 1923 were £14,458,274, and the interest from investments was £684,153. In Scotland the invested funds were £3,870,402 and the interest £182,503.

### Strength of the Voluntary System.

Dr. Menzies contends that the strength of the voluntary hospital system and the real justification for its continuance should be based upon the facts that the immense volume of work is carried out more efficiently and more economically than it can be by any other known method or system. "I am one of those who believe that the public prefer to put their money into a hospital run on the accepted principles of sound finance, rather than a hospital which claims that to be in debt is a virtue instead of a vice."

### Drug Economy.

The approximate estimate of the sum spent annually upon drugs in the provincial hospitals is certainly not less than £200,000. Here, it is suggested, may be some scope for economy :—

> Several years ago, I had occasion to make a detailed enquiry into the costs of the Drug Bill in a number of Tuberculosis Dispensaries, both Voluntary and Municipal. In the course of this enquiry, it very soon became obvious that quite a considerable amount of unnecessary and extravagant expenditure was taking place upon drugs, without any real benefit to the patients, and here again, it became possible to effect great economies without any sacrifice of efficiency, in fact, rather the contrary. Formerly many medical practitioners bought and dispensed their own drugs. In more recent years, the practice of dispensing by medical practitioners has greatly diminished and the vast majority of practitioners prescribe but do not dispense. The change of practice must have resulted in the practitioners generally becoming less and less acquainted with the cost of drugs in pounds, shillings and pence. Without labouring the matter any further, therefore, I should like to suggest to Hospital Boards of Management and their Honorary Medical Staff that they should, where they have not already done so, appoint a Sub-Committee of their Medical Staff to investigate this question. Might I also add the following further suggestions? That the Sub-Committee should call for a number of prescriptions at random from the Wards and Out-patient Department. That the Dispenser should then be asked to price out the cost of each of these prescriptions at so much per bottle of, say, eight or twelve ounces. That, after pricing, the prescriptions should then be examined in detail by the Medical Staff concerned, with a view particularly to determining whether there are any ingredients in each prescription which might be either omitted altogether, or replaced by other ingredients of equal therapeutic value, but costing less.

